# Validation of a questionnaire for assessing household vulnerability to climate change and health among small island communities

**DOI:** 10.3389/fpubh.2025.1593880

**Published:** 2025-06-06

**Authors:** Raheel Nazakat, Mohd Faiz Ibrahim, Fadly Syah Arsad, Noraishah Mohammad Sham, Nik Muhammad Nizam Nik Hassan, Nadia Mohamad, Siti Aishah Rashid, Wan Rozita Wan Mahiyuddin, Rohaida Ismail

**Affiliations:** ^1^Environmental Health Research Centre, Institute for Medical Research, National Institutes of Health, Ministry of Health Malaysia, Shah Alam, Selangor, Malaysia; ^2^Lembah Pantai District Health Office, Kuala Lumpur and Putrajaya Health Department, Kuala Lumpur, Malaysia; ^3^SEAMEO TROPMED Network Malaysia, Institute for Medical Research, National Institutes of Health, Ministry of Health Malaysia, Shah Alam, Selangor, Malaysia

**Keywords:** survey instrument, environmental exposure, susceptible population, household resilience, environmental justice

## Abstract

**Introduction:**

Small island communities in tropical regions are vulnerable to the impacts of climate change. However, there is a lack of a comprehensive tool to assess their health vulnerability, particularly at the household level. This study addresses this gap by developing and validating a questionnaire to evaluate household vulnerability to climate change and health in these communities.

**Materials and methods:**

The questionnaire was constructed in three phases: questionnaire development, validity assessment, and pilot testing. It was developed using a comprehensive framework that incorporated three key dimensions of vulnerability: exposure, sensitivity, and adaptive capacity.

**Results:**

Content validity, evaluated by a panel of experts, demonstrated excellent item-level and scale-level validity indices with S-CVI/UA and S-CVI/Ave of 0.89 and 0.98, respectively. Pilot testing conducted in Carey Island identified 13.5% of households as highly vulnerable. Key contributing factors include high exposure to drought and shoreline erosion, limited access to healthcare, insufficient financial resources, lack of elevated housing structures, and inadequate community engagement and adaptive behavior.

**Discussion:**

The validated tool provides a reliable and context-specific instrument for identifying vulnerable households, enabling policymakers and practitioners to design tailored interventions. This tool provides a structured and evidence-based approach for assessing vulnerability, supporting more effective planning and resilience-building in small island communities facing climate-related health risks.

## 1 Introduction

Although small island communities contribute minimally to climate change, they are particularly vulnerable to the adverse effects of climate change due to their geographic isolation, limited resources, and reliance on external trade and climate-sensitive sectors such as agriculture, fishing, and tourism (1, 2). The impacts of climate change on these communities extend beyond ecosystems and environmental degradation, posing significant threats to socioeconomic stability and public health. Previous research highlights, if the 1.5°C global temperature threshold is exceeded, the small island communities will suffer significant social displacement, economic losses, environmental degradation, and public health challenges (1). Despite producing minimal greenhouse gas emissions, small island communities will bear a disproportionate burden from the damaging impacts of climate change.

In order to understand the extent to which these communities will be affected by climate change and explore potential adaptation strategies, it is imperative to conduct a vulnerability assessment. Vulnerability is defined by the Intergovernmental Panel on Climate Change (IPCC) as the extent to which a system is susceptible to, or unable to cope with, the adverse effects of climate change, including climate variability and extremes (3). Nor Diana et al. (4) define vulnerability as the interplay between (i) internal factors, such as sensitivity and adaptive capacity, which underpin a community’s ability to anticipate, manage, and recover from climate impacts, and (ii) external factors, such as exposure to climate change.

Most vulnerability assessments use an indicator-based approach, which involves specific and measurable use of indicators to assess and quantify the various dimensions of vulnerability (4). The study by Nur Diana et al. (4) utilizes the LVI-IPCC index which combines the livelihood vulnerability index (LVI) and IPCC framework on vulnerability. The LVI-IPCC can be applied broadly, but usually requires further modification to capture specific communities. As such, other studies have applied different variations of the theoretical framework that consisted of exposure, sensitivity, and adaptive capacity indicators (5–10). Since methods of vulnerability measurement are context-dependent, the choice of indicators varies according to regions and situations.

Ehsan et al. (6) which studied household vulnerability in the coastal areas of Selangor, Malaysia, developed a conceptual framework focusing on coastal communities, addressing the specific issues faced by the population living in coastal areas, such as shoreline erosion and coastal flooding, however it did not address specific problems faced by small island communities such as freshwater disparity. As a result, previous assessment tools have been inadequate in capturing the unique and multifaceted public health challenges faced by small island communities, and the lack of standardized, context-specific questionnaires hinders the collection of accurate, comparable, and actionable data. Without these tools, policymakers and stakeholders face challenges in developing targeted interventions that effectively mitigate risks and enhance the resilience of these communities.

Despite the urgent need to address community vulnerabilities, there is a notable absence of standardized tools designed to assess household vulnerability to climate change and health in small island contexts. Therefore, this paper aims to fill this critical gap by validating a newly developed questionnaire designed to evaluate household vulnerability to climate change and health, specifically among small island communities. The validation process employs qualitative and quantitative methods, including expert reviews, content validation, and pilot testing, to establish the questionnaire’s validity. The questionnaire incorporates a wide range of indicators from three dimensions: (a) exposure to climate hazards, (b) sensitivity of households to these hazards, and (c) adaptive capacity, thus providing a nuanced understanding of the specific vulnerabilities faced by the island communities thereby informing targeted interventions and policy decisions.

## 2 Methods

**Figure 1:**
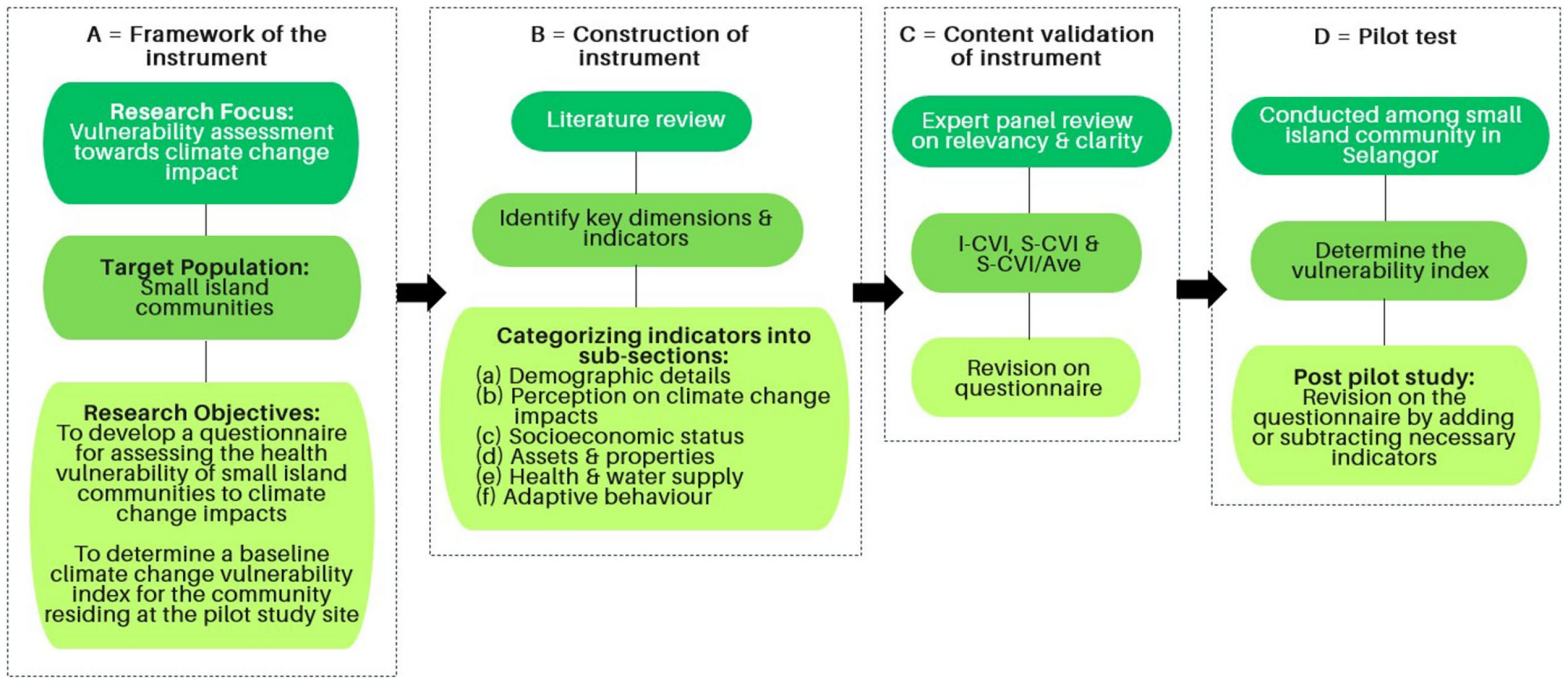
Development process of a climate change vulnerability assessment questionnaire for small island communities.

Developing a questionnaire involves several key steps to ensure it effectively collects all relevant and valuable information. In this study, three phases, as presented in Figure 1, were undertaken to ensure the validity of the findings.

### 2.1 Phase 1: questionnaire development

The present cross-sectional study was conducted from August to December 2023. In light of the limited of a validated questionnaire for evaluating vulnerability to climate change and health, a new questionnaire tool was developed *de novo* by synthesizing existing literature and employing an integrative framework from three dimensions of vulnerability: (a) exposure to climate-related hazards; (b) sensitivity based on demographic, socio-economic, and health factors; and (c) adaptive capacity relating to available resources and behavioral adaptations to mitigate with impacts of climate change. A consensus of the panel identified the components of the dimension and its indicators through a review of published articles using the PubMed and Google Scholar databases, focusing on search terms such as ‘vulnerability and adaptation,’ ‘vulnerability assessment,’ ‘health vulnerability,’ ‘community,’ and ‘climate change.’ The questions were generated for each of the listed indicators, ensuring that they could be used to measure each component, thus the dimension of vulnerability. The questions were distributed accordingly into six focus areas: (a) General information and demographic details of the household head and family members; (b) Perception on climate change impacts; (c) Socioeconomic status; (d) Assets and properties; (e) Health and water supply; and (f) Adaptive behavior toward climate change impact. Then, the content validity of the questions was assessed by individual experts to evaluate the content relevancy and clarity. Finally, the validated questionnaire was pilot-tested among the community residing in the village along the coastal area of Carey Island, Selangor.

### 2.2 Phase 2: assessing the content validity of questionnaire

The content validity of the questionnaire was evaluated using a quantitative scoring system by a panel of six experts (four public health professionals, one academician, and one statistician), all of whom had over 10 years of experience in their respective fields. The experts rated the relevance and clarity of each questionnaire item using a four-point ordered Likert scale: (1) Item is not relevant/clear; (2) Item requires some revision; (3) Item is relevant/clear but requires minor revision; and (4) Item is highly relevant/clear to the measured domain.

The content validity index (CVI), a widely recognized measure of validity (11), was computed based on the ratings provided by the expert panels. Two types of CVIs were calculated: (1) item-level CVI (I-CVI) and (2) scale-level CVI (S-CVI). As part of the statistical method used, the four-point Likert scale responses were dichotomized to enable quantitative analysis. Ratings of 3 (relevant/clear but requires minor revision) and 4 (highly relevant/clear) were scored as 1, indicating the item was considered content valid. Ratings of 1 (not relevant/clear) and 2 (requires some revision) were recorded as 0, indicating that the item was not considered sufficiently relevant or clear to be retained without substantial modification.

The I-CVI for each item was determined by dividing the number of experts who regarded it as relevant by the total number of experts (12), with scores ranging from 0 to 1. According to (13), an I-CVI score of 0.83 or greater is deemed relevant for a panel of six experts, whereas scores ranging from 0.50 and 0.82 indicate the need for revision, and scores below 0.50 imply that the item should be removed.

The S-CVI was determined using two methods: (1) universal agreement among the experts (S-CVI/UA), and (2) average content validity index (S-CVI/Ave). The S-CVI/UA was determined as the proportion of items unanimously rated as relevant (score of one), while the S-CVI/Ave was determined by averaging the I-CVI scores across all items. Excellent content validity is indicated by an S-CVI/UA of 0.8 or higher, and an S-CVI/Ave of 0.9 or higher (11).

### 2.3 Phase 3: pilot testing

A pilot test was conducted in November 2023 among residents of Kampung Melayu, a coastal community located on Carey Island, Selangor. Carey Island shares key characteristics with other small islands, such as being low-lying and exposed to threats like sea level rise, shoreline erosion, and saltwater intrusion, making it a relevant site to study small island vulnerabilities to climate change (14, 15). The study objectives and procedures were explained to participants, and written informed consent was obtained prior to their voluntary participation. A total of 37 eligible heads of households participated as respondents. Data collected during the pilot were utilized to construct a composite vulnerability index (CVI), categorized into four levels: low, moderate, high, and very high, based on quarterly percentile distribution. Respondents also provided feedback on the clarity and comprehensibility of the survey questions. Following the pilot test, the investigators conducted a review to identify and implement potential refinements to the assessment tools based on the findings and participant feedback.

## 3 Results

### 3.1 Phase 1: questionnaire development

**Figure 2:**
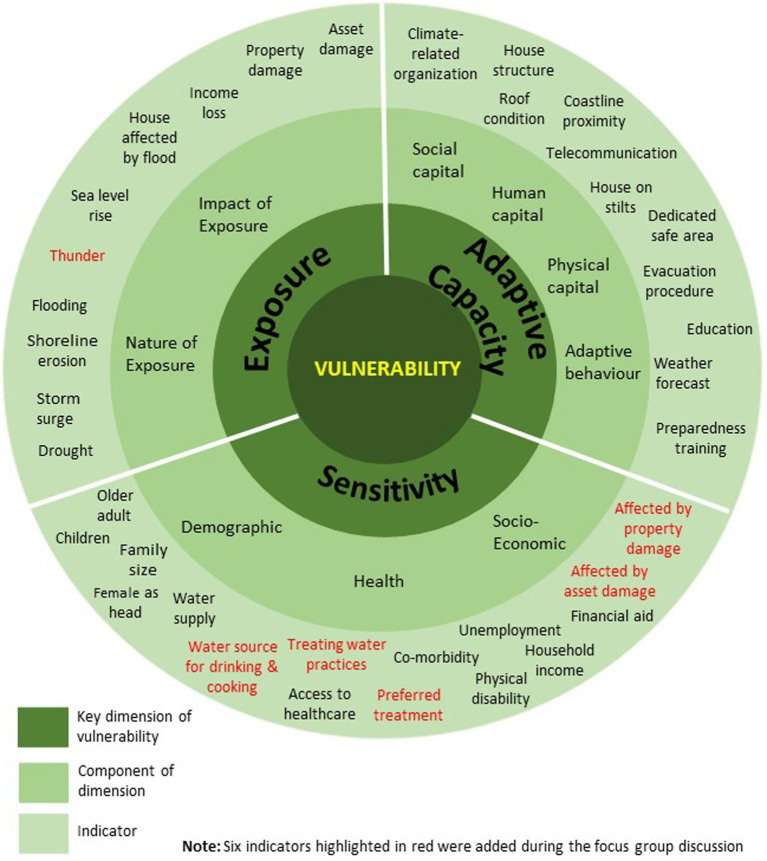
Climate change vulnerability assessment indicators based on exposure, sensitivity, and adaptive capacity.

From the literature review, 26 published articles were identified, which helped pinpoint the key dimensions of vulnerability and the most relevant indicators for the study’s objectives. There were 31 indicators identified through the literature review, and the list was summarized in Appendix A. Subsequently, a focus group discussion (FGD) involving all investigators was held in September 2023 to refine the vulnerability dimensions and indicators of climate change and health. During the FGD, six more indicators were added, and the components of each dimension, along with their respective indicators, were summarized in Figure 2. The identified indicators were then used to guide the development of the study questionnaire, which was designed w+ith all questions presented in Malay language.

The descriptive analysis of the questionnaire domains revealed varied levels of responses across the different areas assessed. In the Perception on Climate Change domain, which consisted of nine indicators, respondents recorded a median score of 4, with a mean of 4.27 and a standard deviation of 1.500, indicating a generally high level of awareness and perception of climate change issues, accompanied by moderate variability among the respondents. In contrast, the Socioeconomic Status domain, with two indicators, showed a median score of 1, a mean of 0.784, and a standard deviation of 0.703, suggesting that socioeconomic challenges were present but varied less widely across participants. The Assets and Properties domain, which included five indicators, had a median of 1, a mean of 1.486, and a standard deviation of 1.029, implying that ownership of assets and properties was relatively limited but demonstrated notable variability among households. Similarly, the Health and Water Supply domain, comprising five indicators, recorded a median of 1, a mean of 0.946, and a standard deviation of 0.226, reflecting generally low concerns in this area with very little variability among responses. Finally, in the Adaptive Behavior domain, which included four indicators, respondents also recorded a median of 1, a mean of 1.00, and a standard deviation of 0.986, indicating a modest level of adaptive actions with some degree of variation across the sample (Table 1).

**Table 1:** Descriptive statistics of the domain in the questionnaire.

Domain	Number of indicators	Median	Mean	Standard deviation
Perception on climate change	9	4	4.27	1.500
Socioeconomic status	2	1	0.784	0.703
Assets and properties	5	1	1.486	1.029
Health and water supply	5	1	0.946	0.226
Adaptive behavior	4	1	1	0.986

### 3.2 Phase 2: assessing the content validity of questionnaire

A total of 40 questions were developed, derived from the 37-climate change vulnerability assessment indicators, and were rigorously evaluated for content validation by the expert panels (Table 2).

**Table 2:** The item-level content validity index (I-CVI) scores for relevancy and clarity.

Criteria	Item number	Total items	Researcher consideration
Items with an I-CVI of 1.00 for both relevancy and clarity	1–5, 11, 15–16, 18–28, 30, 32–36, 38–40	28	Retained all items
Items with an I-CVI of 1.00 for either relevancy or clarity, and 0.83 for the other	17, 29, 31, 37	4	Retained all items and refined them accordingly
Items with an I-CVI of 1.00 for either relevancy or clarity, and 0.67 for the other	6–10, 12–14	8	Retained all items and refined them accordingly

All the items, especially those with I-CVI scores of 0.83 and 0.66, were retained after being refined based on expert comments, recommendations, and discussion among the investigators. The 40-item questionnaire’s S-CVI/UA scores were 0.89 for relevancy and 0.82 for clarity, while the S-CVI/Ave scores were 0.98 for relevancy and 0.94 for clarity. These results indicate that the questionnaire demonstrates excellent content validity.

### 3.3 Phase 3: pilot testing

The majority heads of 37 households in this study were male (89.2%), while female respondents constituted 10.8%. Regarding age distribution, almost half of the participants (48.7%) were aged more than 55 years, followed by 24.3% aged between 46 and 55 years, 16.2% aged 35 to 45 years, and 10.8% aged less than 35 years. In terms of educational background, 43.2% had completed primary education, 37.9% had attained secondary education, and 16.2% had tertiary education. A small proportion (2.7%) reported no formal education.

For marital status, the vast majority were married (97.3%), with only 2.8% reported as single. Employment status indicated that 81.1% were employed, while 18.9% were unemployed. Regarding household size, 59.5% of households had fewer than five members, whereas 40.5% had more than five members. In terms of income, 78.4% of respondents reported earning less than RM4,850 monthly, while 21.6% reported earning more than RM4,850. Furthermore, 67.6% of the respondents reported having comorbidities, compared to 32.4% who did not report any (Table 3).

**Table 3:** Demographic characteristics of head of households.

Item	Number	Percentage (%)
Gender		
Male	33	89.2
Female	4	10.8
Age		
Less than 35 years old	4	10.8
35–45 years old	6	16.2
46–55 years old	9	24.3
More than 55 years old	18	48.7
Level of education		
Primary	16	43.2
Secondary	14	37.9
Tertiary	6	16.2
No formal education	1	2.7
Marital status		
Single	1	2.8
Married	36	97.3
Employment status		
Employed	30	81.1
Unemployed	7	18.9
Number of households		
Less than 5	22	59.5
More than 5	15	40.5
Income		
Less than RM4,850	29	78.4
More than RM4,850	8	21.6
Comorbidity		
Yes	25	67.6
No	12	32.4

**Figure 3:**
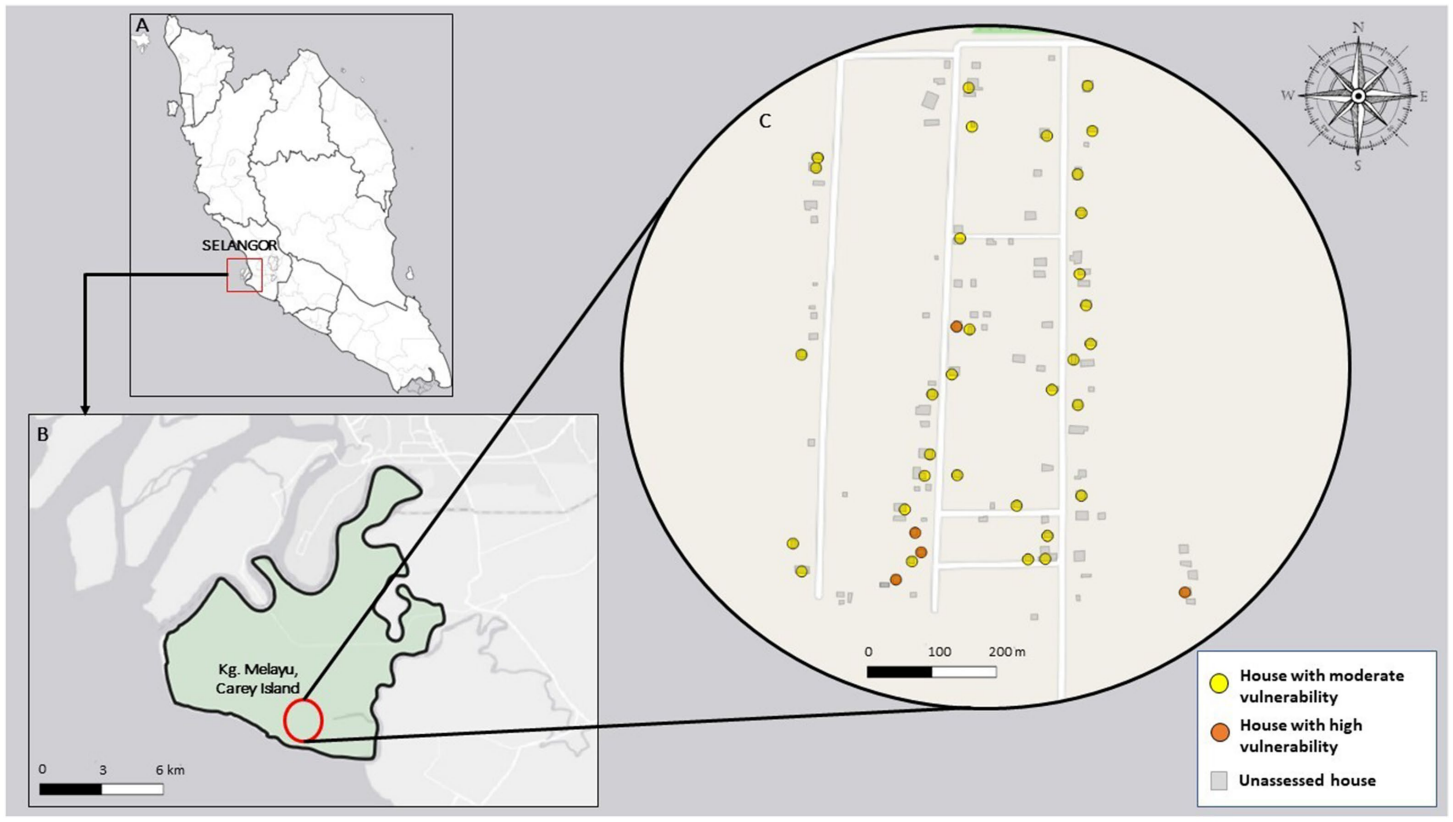
Spatial distribution of household’s vulnerability to climate change in Kampung Melayu, Carey Island. **(A)** represents the map of Peninsular Malaysia with a red box marking the location of Carey Island, Selangor. **(B)** represents a zoomed-in map of Carey Island, showing the study site (Kg. Melayu) marked with a red circle. **(C)** represents a detailed map of Kg. Melayu shows individual households’ vulnerability levels.

Figure 3 illustrates the spatial distribution of household vulnerability to climate change in Kampung Melayu, Carey Island. The spatial distribution reveals varying levels of vulnerability influenced by localized factors, as indicated in the vulnerability assessment. Most households (86.5%) exhibited moderate vulnerability, represented by the predominance of yellow markers. A smaller proportion (13.5%) demonstrated high vulnerability, with a cluster of four households located in the southern regions of the village near the coastal fringe.

**Figure 4:**
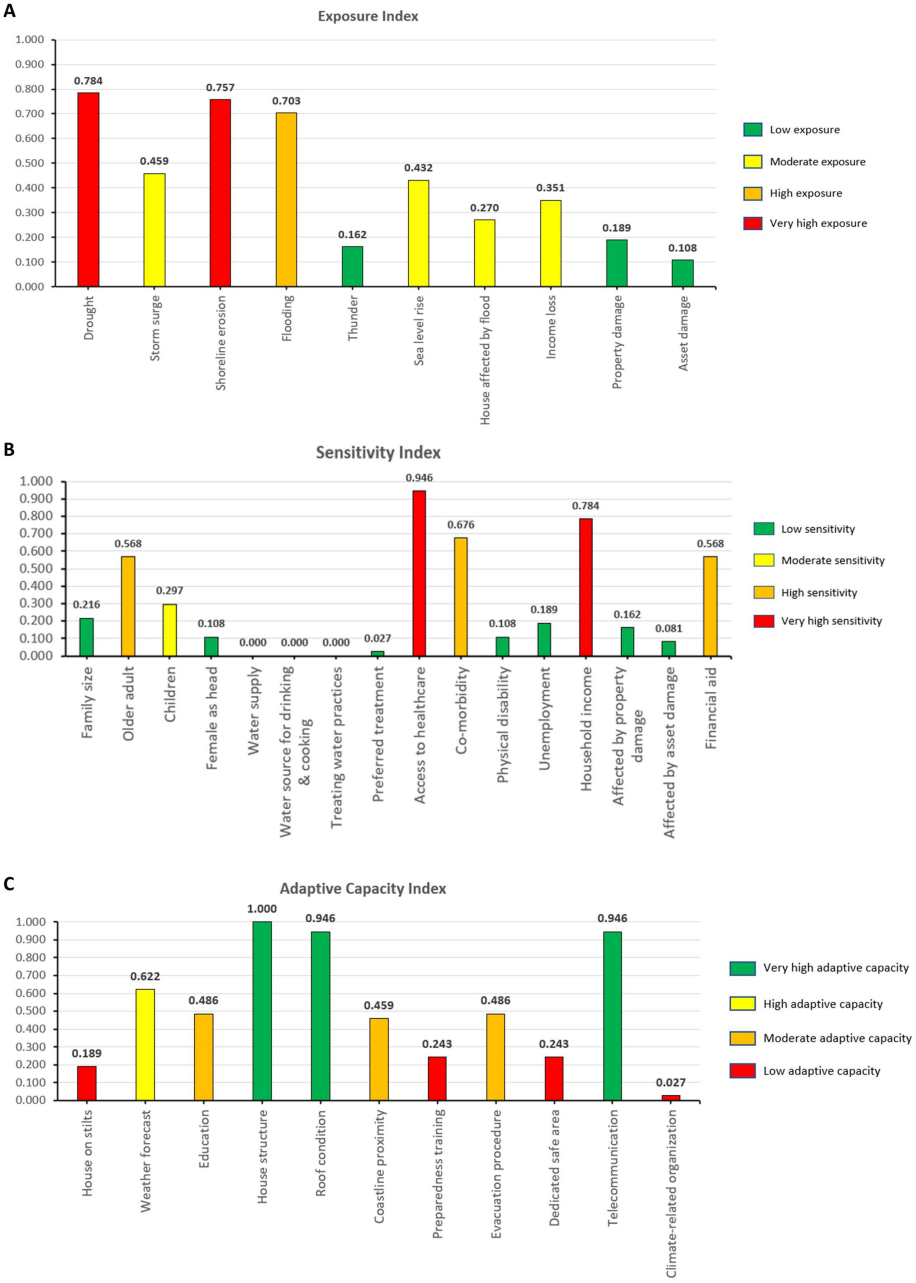
Contributing factors of **(A)** exposure index, **(B)** sensitivity index, and **(C)** adaptive capacity index for climate change vulnerability assessment.

A detailed analysis of the findings is summarized in Figure 4. Key findings indicate significant exposure to drought (index = 0.784) and shoreline erosion (index = 0.757), with flooding (index = 0.703) also posing a considerable threat. Sensitivity analysis revealed a very high index among households with limited access to healthcare services (index = 0.946) and low incomes (index = 0.784). Furthermore, households with at least one member with co-morbidity (index = 0.676), households with at least one older adult member (index = 0.568), and households with limited financial aid (index = 0.568) were identified with a high sensitivity index. The adaptive capacity index was found to be low because of the following reasons: (a) lack of access to dedicated safe areas (index = 0.243), (b) inadequate preparedness training (index = 0.243), (c) houses were built on stilts (index = 0.189), and (d) lack of involvement in climate-related organizations (index = 0.027).

The respondent’s scores across different questionnaire dimensions can be leveraged to assess the vulnerability and identify factors contributing to household-level vulnerability. For example, an anonymous respondent (R036) in the pilot study recorded a CVI of 0.606 (high vulnerability), an exposure index of 0.500 (moderate exposure), a sensitivity index of 0.500 (moderate sensitivity), and an adaptive capacity index of 0.182 (low adaptive capacity). Compared to its exposure and sensitivity, the household’s significantly lower adaptive capacity resulted in a higher overall vulnerability. A further observation of the findings for R036 identified several contributing factors to the vulnerability, including inadequate financial capacity, low education attainment, living close to the coastline, limited access to healthcare, having family members with co-morbidity and physical disability, and lack of adaptive behavior.

## 4 Discussion

This study presents the development and validation of a novel questionnaire designed to assess household vulnerability to climate change and health in small island communities. To our knowledge, this is the first tool explicitly tailored for use among communities residing in tropical island settings, where unique environmental and socio-economic challenges often exacerbate vulnerability. The development process included a comprehensive literature review, performing validity testing, and conducting pilot studies. Our approach ensured a robust foundation, identifying three key dimensions of vulnerability: exposure, sensitivity, and adaptive capacity. These dimensions align with existing vulnerability frameworks (16), enhancing the questionnaire’s relevance and applicability. In epidemiological research, using a validated questionnaire is essential to ensure the accurate measurement of the intended variables, as employing a non-validated instrument may introduce measurement errors and compromise the validity of the outcomes (17). In particular, the availability of a validated instrument is crucial for designing policies to improve the resilience and health adaptation of small island communities toward climate change impacts.

Validation results indicate that the questionnaire has high content validity for relevancy and clarity. Various validity statistics confirmed the excellent content validity, including I-CVI, S-CVI/UA, and S-CVI/Ave. The items on the questionnaire strongly represented the thematic dimensions. Through iterative refinement and pilot testing, the final version emerged as a reliable tool tailored to the study’s objectives. This process aligns with the framework described in the foundational work by Lawshe (18) and has been similarly applied in recent studies (19, 20).

The pilot testing phase involved administering the questionnaire to a representative sample of small island communities, allowing for real-world evaluation and necessary adjustments. Feedback from participants and analysis of pilot data led to refinements in wording, item clarity, and relevance, ensuring that the tool is both user-friendly and scientifically robust. This rigorous development process validates the questionnaire and ensures practical applicability in assessing vulnerability in small island communities. Unlike other household vulnerability assessment tools designed for urban (21, 22) or coastal communities (6, 23), this questionnaire was specifically developed for communities in tropical island settings, with focus on both climate change impacts and health vulnerabilities. Small island communities face distinct environmental and socio-economic challenges, such as heightened exposure to natural disasters (24), limited resources and facilities, and inadequate financial capacity (2), all amplifying their vulnerability to climate change. While certain existing tools focus on single aspects, such as adaptive capacity (25, 26), this questionnaire offers a more holistic evaluation by integrating all three core dimensions: exposure, sensitivity, and adaptive capacity. Supporting this approach, a study in Indonesia’s Mepar and Baran Islands reported high concern among residents regarding sea-level rise and extreme weather events, reflecting elevated exposure (27). Moreover, their findings emphasized how socioeconomic factors, including age, education, place of birth, and trade engagement, influence both risk perception and adaptive responses (27), aligning closely with the multidimensional framework underpinning our tool.

The current study found a prevalence of 13.5% for high vulnerability to climate change among the surveyed small island communities. High exposure to prolonged dry spells and coastal erosion was notably prevalent. This finding aligns with previous studies indicating that small island communities are particularly susceptible to these environmental stressors due to their geographic and climatic conditions (28, 29). Small islands often face unique challenges, such as limited land area, high population density, and dependency on natural resources, exacerbating their vulnerability to climate-related impacts. The prevalence of high exposure to temperature fluctuations and coastal erosion underscores the urgent need for targeted interventions to mitigate these specific risks. Despite these high exposure rates, some island inhabitants demonstrated good resilience, characterized by moderate to high adaptive capacity indices (25, 26). This suggests that certain population segments possess the knowledge, resources, and strategies to cope with climate-related stresses. Factors contributing to this resilience may include robust community networks, traditional ecological knowledge, and effective local governance structures that promote adaptive practices. These findings highlight the importance of leveraging community strengths to enhance resilience. However, among those with low adaptive capacity, common factors included limited access to healthcare and low household incomes. In our study, households with incomes less than RM4, 850 (Malaysia’s bottom 40% income category) were particularly vulnerable, reflecting the direct correlation between economic stability and adaptive capacity. Limited financial resources can restrict access to critical adaptation measures, such as improved housing, diversified livelihoods, and educational opportunities (6, 35). Similarly, restricted access to healthcare services impedes addressing health-related impacts of climate change, such as increased incidence of heat-related illnesses and vector-borne diseases.

These issues reflect broader socioeconomic challenges highlighted in prior research, where limited financial resources and healthcare access significantly impair community resilience to climate change. Previous studies have consistently shown that socioeconomic factors, such as income, education, and health, are crucial determinants of a community’s ability to adapt to climate change (7, 30–32). For example, low income constrains the ability to invest in adaptation measures, such as elevating residences above the ground, to safeguard properties and assets (7). Moreover, the intersection of economic and health disparities creates compounded vulnerabilities that can hinder effective adaptation. For instance, a lack of financial resources can prevent households from accessing adequate healthcare, which is essential for maintaining the health and well-being necessary to engage in adaptive activities. This intersectionality underscores the need for comprehensive adaptation strategies that effectively address economic and health dimensions to enhance community resilience.

The findings from this study offer critical insights to inform the development of climate adaptation policies and interventions for small island communities. By identifying households with high vulnerability to climate-related risks, the validated questionnaires provide a practical tool for targeting adaptation measures where they are most needed. These may include strengthening coastal protection, improving access to healthcare and early warning systems, enhancing water security, and supporting climate-resilient livelihoods (33). Addressing adaptive capacity deficits, such as limited financial resources and weak community engagement, can substantially enhance resilience at both household and community level. Furthermore, integrating this tool into national and local adaptation planning supports ongoing monitoring and aligns with global frameworks such as Paris Agreement (34).

### 4.1 Strengths and limitations

The primary strength of this study is the systematic and comprehensive approach to developing and validating the questionnaire. The rigorous process ensured that the tool is both reliable and applicable to small island settings. Additionally, the pilot testing provided valuable insights into the questionnaire’s real-world application and potential impacts. However, there are limitations to consider. While adequate for initial validation, the sample size for pilot testing may not fully capture the diversity of small island communities. Further research with larger and more varied samples would be beneficial. Furthermore, test–retest reliability was not assessed due to resource constraints. Additionally, while the tool was designed specifically for households in tropical islands, its applicability to other regions requires further investigation and possible adjustments.

### 4.2 Recommendations

Future research should aim to refine the questionnaire further and validate its applicability across different island settings. Expanding the sample size and including diverse island communities, such as those of different geographical regions, will help understand the tool’s broader applicability. Additionally, future studies should assess the tool’s test–retest reliability and, where applicable, inter-rater reliability to further establish its consistency over time and across different users. Furthermore, future studies should incorporate inferential statistical analyses to evaluate significant differences across household vulnerability levels and between different vulnerability dimensions. On top of that, further study should explore the application of this tool in other vulnerable settings and adapt it based on the unique characteristics of different communities, ensuring its effectiveness in supporting evidence-based decision-making.

## 5 Conclusion

This study successfully developed and validated a questionnaire to assess household vulnerability to climate change and health in small island communities in a tropical country. The instrument demonstrated excellent content validity, confirming its robustness and suitability for this unique context. By providing a reliable tool for identifying and understanding vulnerability, this study lays the groundwork for future research and policy development to reduce vulnerability and enhance resilience in small island communities.

## Data Availability

The datasets presented in this article are not readily available because the dataset is restricted under ethical approval and informed consent, limiting access to the research team only. Requests to access the datasets should be directed to raheel@moh.gov.my.

## Supplementary material

The Supplementary material for this article can befound online at: https://www.frontiersin.org/articles/10.3389/fpubh.2025.1593880/full#supplementary-material
