# Supplementary material for: Validation of a questionnaire for assessing household vulnerability to climate change and health among small island communities
Source: Front Public Health. 2025 Jun 6;13:1593880. doi: 10.3389/fpubh.2025.1593880 (PMC12179156; doi:10.3389/fpubh.2025.1593880)
Supplement: Supplementary file 1 [file Table_1.docx]

**Appendix A**

**Table 1:** Literature review of the list of indicators for the key dimension exposure

| **No.** | **Author & Year** | **Drought** | **Heavy rain/Storm surge** | **Shoreline erosion** | **Flooding** | **Sea level rise** | **House affected by flood** | **Income loss** | **Property damage** | **Asset damage** |
| --- | --- | --- | --- | --- | --- | --- | --- | --- | --- | --- |
| 1 | Nguyen et al., 2017 | √ | √ | √ | √ | √ |  |  |  |  |
| 2 | Martin & Gassala, 2020 |  | √ | √ |  |  |  |  |  |  |
| 3 | Ashikin et al., 2021 |  | √ |  | √ |  | √ | √ |  |  |
| 4 | Kuchlicke et al., 2011 |  |  |  | √ |  | √ |  |  |  |
| 5 | Majid et al., 2019 |  |  |  | √ |  | √ | √ |  | √ |
| 6 | Diana et al., 2019 |  |  |  | √ |  |  | √ |  |  |
| 7 | Hahn et al., 2009 | √ | √ |  | √ |  |  |  |  |  |
| 8 | Adu et al., 2018 | √ | √ |  | √ |  |  |  |  |  |
| 9 | Madhuri et al., 2015 |  |  |  |  |  |  |  |  |  |
| 10 | Panthi et al., 2015 | √ | √ |  | √ |  |  |  |  |  |
| 11 | Brechwald et al., 2015 |  |  |  | √ |  |  |  |  |  |
| 12 | Carvalhaes & Omitaomu, 2017 |  |  |  |  |  |  |  |  |  |
| 13 | Cooley et al., 2012 | √ |  |  | √ |  |  |  |  |  |
| 14 | Nong et al., 2020 | √ | √ |  | √ |  |  |  |  |  |
| 15 | Abdul-Razak & Kruse, 2017 |  |  |  |  |  |  |  |  |  |
| 16 | Brodie et al., 2006 |  |  |  |  |  |  |  |  |  |
| **No.** | **Author & Year** | **Drought** | **Heavy rain/Storm surge** | **Shoreline erosion** | **Flooding** | **Sea level rise** | **House affected by flood** | **Income loss** | **Property damage** | **Asset damage** |
| 17 | Huynh & Stringer, 2018 |  | √ |  |  |  |  |  |  |  |
| 18 | Ehsan et al., 2022 | √ | √ | √ | √ |  |  |  | √ | √ |
| 19 | Truong et al., 2022 |  |  |  | √ |  | √ |  |  | √ |
| 20 | Caceres et al., 2021 | √ | √ |  | √ |  |  |  |  |  |
| 21 | Mwangi et al., 2020 | √ | √ |  |  |  |  |  |  |  |
| 22 | Castro et al., 2019 |  |  |  | √ |  |  |  |  |  |
| 23 | Ali et al., 2022 | √ | √ |  | √ |  |  |  |  |  |
| 24 | Zacarias, 2019 | √ | √ |  |  |  |  |  |  |  |
| 25 | Piya et al., 2012 | √ | √ |  |  |  |  |  |  |  |
| 26 | Fernandez & Golubiewski, 2019 | √ | √ |  |  | √ |  |  |  |  |

**Table 2:** Literature review of the list of indicators for the key dimension sensitivity

| **No.** | **Author & Year** | **Elderly** | **Children** | **Family size** | **Female as head** | **Water supply** | **Access to healthcare** | **Co-morbidity** | **Physical disability** | **Unemployment** | **Household income** | **Financial aid** |
| --- | --- | --- | --- | --- | --- | --- | --- | --- | --- | --- | --- | --- |
| 1 | Nguyen et al., 2017 | √ | √ |  | √ | √ |  |  |  |  |  |  |
| 2 | Martin & Gassala, 2020 |  |  |  |  |  |  |  |  |  |  |  |
| 3 | Ashikin et al., 2021 |  |  | √ | √ | √ | √ | √ |  |  | √ | √ |
| 4 | Kuchlicke et al., 2011 | √ | √ | √ | √ |  |  | √ | √ | √ | √ |  |
| 5 | Majid et al., 2019 |  |  | √ | √ | √ | √ | √ |  |  | √ | √ |
| 6 | Diana et al., 2019 |  |  |  | √ | √ | √ |  |  |  | √ | √ |
| 7 | Hahn et al., 2009 |  |  |  | √ | √ | √ | √ |  |  |  |  |
| 8 | Adu et al., 2018 |  |  |  | √ | √ | √ | √ |  |  |  |  |
| 9 | Madhuri et al., 2015 |  |  |  | √ | √ |  | √ |  |  |  |  |
| 10 | Panthi et al., 2015 | √ | √ |  | √ |  | √ | √ |  |  |  |  |
| 11 | Brechwald et al., 2015 | √ | √ |  |  |  |  |  |  |  | √ |  |
| 12 | Carvalhaes & Omitaomu, 2017 | √ | √ |  | √ |  |  |  | √ | √ | √ |  |
| 13 | Cooley et al., 2012 | √ | √ |  | √ |  |  | √ | √ |  | √ |  |
| 14 | Nong et al., 2020 |  |  |  | √ | √ |  | √ |  |  |  |  |
| 15 | Abdul-Razak & Kruse, 2017 |  |  |  |  |  |  |  |  |  |  |  |

| **No.** | **Author & Year** | **Elderly** | **Children** | **Family size** | **Female as head** | **Water supply** | **Access to healthcare** | **Preferred treatment** | **Co-morbidity** | **Physical disability** | **Unemployment** | **Household income** | **Financial aid** |
| --- | --- | --- | --- | --- | --- | --- | --- | --- | --- | --- | --- | --- | --- |
| 16 | Brodie et al., 2006 |  |  |  |  |  |  |  |  |  |  |  |  |
| 17 | Huynh & Stringer, 2018 |  |  |  |  |  |  |  | √ |  |  | √ |  |
| 18 | Ehsan et al., 2022 | √ | √ | √ | √ | √ |  |  |  |  | √ | √ |  |
| 19 | Truong et al., 2022 |  |  |  |  | √ | √ |  |  |  |  | √ |  |
| 20 | Caceres et al., 2021 | √ | √ | √ | √ | √ | √ |  | √ | √ | √ |  |  |
| 21 | Mwangi et al., 2020 |  |  |  |  | √ |  |  |  |  |  | √ |  |
| 22 | Castro et al., 2019 | √ | √ |  |  | √ | √ |  |  |  |  |  |  |
| 23 | Ali et al., 2022 |  |  | √ |  | √ | √ |  | √ |  |  |  |  |
| 24 | Zacarias, 2019 |  |  | √ | √ | √ | √ |  | √ |  |  | √ |  |
| 25 | Piya et al., 2012 |  |  |  | √ |  |  |  |  |  |  | √ |  |
| 26 | Fernandez & Golubiewski, 2019 | √ | √ |  |  |  |  |  |  |  | √ | √ |  |

**Table 3:** Literature review of the list of indicators for the key dimension adaptive capacity

| **No.** | **Author & Year** | **Climate -related organization** | **House structure** | **Roof condition** | **Coastline proximity** | **Telecommunication** | **House on stilts** | **Dedicated safe area** | **Evacuation procedure** | **Education** | **Weather forecast** | **Preparedness training** |
| --- | --- | --- | --- | --- | --- | --- | --- | --- | --- | --- | --- | --- |
| 1 | Nguyen et al., 2017 |  |  |  |  |  |  |  |  |  |  |  |
| 2 | Martin & Gassala, 2020 | √ |  | √ |  |  |  |  |  |  |  |  |
| 3 | Ashikin et al., 2021 |  | √ |  |  | √ | √ | √ |  | √ |  |  |
| 4 | Kuchlicke et al., 2011 | √ |  |  |  |  |  |  |  | √ |  |  |
| 5 | Majid et al., 2019 | √ | √ |  |  |  | √ |  |  | √ |  |  |
| 6 | Diana et al., 2019 | √ |  |  |  |  |  |  |  | √ |  | √ |
| 7 | Hahn et al., 2009 |  |  |  |  |  |  |  |  | √ |  |  |
| 8 | Adu et al., 2018 |  |  |  |  |  |  |  |  | √ |  |  |
| 9 | Madhuri et al., 2015 |  |  |  |  |  |  |  |  | √ |  |  |
| 10 | Panthi et al., 2015 |  |  |  |  | √ |  |  |  | √ |  |  |
| 11 | Brechwald et al., 2015 |  |  |  |  |  |  |  |  | √ |  |  |
| 12 | Carvalhaes & Omitaomu, 2017 |  |  |  |  | √ |  |  |  | √ |  |  |
| 13 | Cooley et al., 2012 |  |  | √ |  | √ |  |  |  | √ |  |  |
| 14 | Nong et al., 2020 | √ |  |  |  | √ |  |  |  | √ |  |  |
| **No.** | **Author & Year** | **Climate -related organization** | **House structure** | **Roof condition** | **Coastline proximity** | **Telecommunication** | **House on stilts** | **Dedicated safe area** | **Evacuation procedure** | **Education** | **Weather forecast** | **Preparedness training** |
| 15 | Abdul-Razak & Kruse, 2017 | √ |  |  |  | √ |  |  |  | √ |  |  |
| 16 | Brodie et al., 2006 |  |  |  |  | √ |  |  | √ |  |  |  |
| 17 | Huynh & Stringer, 2018 | √ | √ | √ |  | √ |  |  |  | √ |  |  |
| 18 | Ehsan et al., 2022 | √ | √ | √ | √ | √ | √ | √ | √ | √ | √ |  |
| 19 | Truong et al., 2022 | √ |  |  |  | √ |  |  |  | √ |  |  |
| 20 | Caceres et al., 2021 |  |  |  |  |  |  |  |  | √ |  |  |
| 21 | Mwangi et al., 2020 |  | √ |  |  |  |  |  |  |  |  |  |
| 22 | Castro et al., 2019 |  |  |  |  |  |  |  |  | √ |  |  |
| 23 | Ali et al., 2022 |  |  |  |  |  |  |  |  | √ |  |  |
| 24 | Zacarias, 2019 | √ |  |  |  | √ |  |  |  | √ |  |  |
| 25 | Piya et al., 2012 | √ |  |  |  | √ |  |  |  | √ |  | √ |
| 26 | Fernandez & Golubiewski, 2019 |  |  |  |  |  |  |  |  |  |  |  |
